# Development of children’s hymenoptera venom allergy quality of life scale (CHVAQoLS)

**DOI:** 10.1186/2045-7022-3-25

**Published:** 2013-08-01

**Authors:** Ewa Cichocka-Jarosz, Piotr Brzyski, Beata Tobiasz-Adamczyk, Grzegorz Lis, Jacek J Pietrzyk

**Affiliations:** 1 Department of Pediatrics, Polish-American Institute of Pediatrics, Jagiellonian University Medical College, Krakow, Poland; 2 Department of Medical Sociology, Chair of Epidemiology and Preventive Medicine, Jagiellonian University Medical College, Krakow, Poland

**Keywords:** Development, Validity, Reliability, Health-related quality of life, Scale, Hymenoptera venom, Allergy

## Abstract

**Background:**

Venom allergy is a rare but life-threatening disease and may have a considerable impact on the health-related quality of life (HRQoL) of patients, especially children. This paper presents development of the HRQoL scale for children and adolescents with Hymenoptera venom allergy (HVA).

**Methods:**

The study sample consisted of 71 children, born between 1992 and 2000, who presented with a history of insect sting reaction when referred for consultation in the allergy center of Polish-American Children’s Hospital, Krakow, Poland, during the period from 2000 to 2010. The initial pool of 60 items - divided into 6 domains - was prepared. The items with intercorrelations higher than 0.7 were removed from each domain and then principal component analysis was conducted for each domain separately, to provide a one-dimensional subscale for each domain. Reliability of the subscales was assessed using Cronbach alpha coefficient in terms of Classical Test Theory and with rho coefficient in terms of Item Response Theory. The multidimensionality of the scale was tested using multi-trait scaling.

**Results:**

Three to four items from each domain were subsequently selected to constitute six subscales. Rho coefficients for all the subscales reached 0.8, similar results were achieved with the Cronbach alpha coefficients. Multi-trait method showed that the majority of the items indicated stronger correlations with their own subscales than with other subscales, which proves that our constructed subscales measure different dimensions of HRQoL.

**Conclusions:**

The presented scale comprises high validity and reliability subscales measuring six dimensions of HRQoL related to Hymenoptera venom allergy in children and adolescents. Such information may be useful in everyday clinical practice.

## Background

Venom allergy is a rare but life-threatening disease; the awareness of this fact, as well as symptoms of the disease itself, may have a profound impact on health-related quality of life (HRQoL) of affected individuals, especially children. This makes the measurement of HRQoL in this group of patients very important. The first tool for measuring HRQoL in adult patients with allergy to wasp venom – Vespid Quality of Life Questionaire (VQLQ) – was published in 2002 [1]. This index consisted of 14 items, selected on the basis of impact methodology, which, according to the results of content validity analysis, could be divided into four domains: anxiety, caution, limitations, and discomfort. However, the authors presented the scale as a one-dimensional measurement tool, whose score is computed as a mean of its’ items scores [1,2]. Factor analysis was conducted based on the Polish adaptations of VQLQ for adolescents and children with Hymenoptera venom allergy (HVA) - and for parents of those children - supported the results of content analysis. It demonstrated that the index had a 3-factor structures in separate samples including children and adolescents, and a 4-factor structure in the sample of parents, with the 4^th^ factor defined by one item concerning limitations [3,4]. The same authors also showed that each dimension of HRQoL of this group of patients was affected by different determinants [5,6]. This suggested that efforts aiming at developing a scale measuring HRQoL in HVA patients should be placed on a valid measurement of a particular dimension rather than on the importance of particular items, as was done in case of VQLQ.

The method of selection of items employed in the development of VQLQ led to a construction of an index of the most important difficulties in the life of patients with HVA. However, the suggested use of this tool as an unidimensional one leads to a situation where the impact of various kinds of problems on overall perception of quality of life is treated as proportional to the number of items included into a particular domain, and forces an assumption that every item has the same impact on overall HRQoL – neither seem justified. A situation thereby arises wherein the factor structure of the scale differs between samples, with different social or demographic characteristics increasing the weight of the problem and as such a question arises about interpretation of the obtained results and their comparability. HRQoL is – by definition – a multidimensional construct and each of the domains, which are to be taken into account in case of a particular group of patients, should be considered separately [7]. Moreover, according to the reports of Cronbach, defining the tool as unidimensional does not lead to measurement of some abstract latent variable (called HRQoL in this case), but to measurement of the first latent variable of those responsible for the variance of the scale items [8]. Our research has shown that discomfort is actually the variable measured by the VQLQ, because items measuring discomfort defined first dimension of PCA solutions for both Polish VQLQ adaptations [3,4].

On the other hand, measurement of the level of limitations employed in the VQLQ is limited to one question only, hence it seems not to be very reliable, since it allows for very broad understanding of patient’s limitations, and may cause problems when this level differs between various areas of patient’s life. Lesser number of items measuring limitations may also suggest that this dimension of HRQoL is less important than others.

Additionally, among seven items of VQLQ concerning discomfort, five have conditional structure: the respondent scored his/her level of being troubled only if he/she answered “yes” to the filtering question. In such a case, individuals who did not respond to some conditional items are supposed to answer these items with the score equal to the mean of all the answered items (including also anxiety, cautions and limitations). However, it is possible that the patient’s answers to these questions would be different if these situations really concerned him and as such would cause unexpected variance in the omitted items.

The most important doubt concerning the psychometric properties of VQLQ addresses the fact that the scale was validated using an outcome expectation scale, which itself was created to validate this particular tool. The process of validation - included in the same paper - was based exclusively on calculation of Cronbach alpha coefficient [1].

The above-mentioned issues and psychometric properties of Polish adaptations of VQLQ for children and adolescents with HVA encouraged us to develop a scale measuring the HRQoL of this group of patients. Our efforts were directed towards defining the dimensions of HRQoL that would be most important for children and adolescents with HVA, and formulating the items which would be observable indicators of each dimension in a manner so that they could be understood by adolescents (aged 14+) and children (aged under 14), the sample audience of our survey. Similar number of items considered to be the best indicators of the latent variables represented by each dimension of HRQoL were chosen, so that they would be measured with a possibly high validity and reliability. This paper presents the results of development of such a scale.

## Material and methods

### Study sample

The study sample consisted of 71 children, aged 10 to 18, mean age 13.3 years, SD 2.9, who were suspected of having HVA and thus referred to an allergist for consultation, at Polish-American Children’s Hospital, Kraków, Poland. Consultations were held during the period from 2000 to 2010. Demographic and clinical characteristics of the sample is presented in Table 1. The written permission from patients’ parents was obtained. The study took place from May to July 2010. The study was approved by the Jagiellonian University Bioethical Committee (KBET No 67/L/2007, June 28, 2007).

**Table 1:** Demographic and clinical characteristics of children under study

		**n**	**%**
Gender	boys	51	71,8
	girls	20	28,2
Mueller’s grade	I	10	14,1
	II	15	21,1
	III	24	33,8
	IV	22	31,0
Place of residence	town	28	39,4
	village	43	60,6
Venom	bee	34	47,9
	wasp	32	45,1
	bee and wasp	5	7,0
Total		71	100

### Methods

Development of the scale proceeded in the following stages:

Stage 1) Based on the existing scales, analysis of literature, and contacts with children during allergist consult, a baseline pool of 60 items divided into six sets of items (called domains in the further part of the paper) was generated. A pediatrician, a pediatric psychologist, a sociologist and a linguist all reviewed the questionnaire to assess it for clarity and ease of use by children. The particular domains included anxiety about insects and a possibility of being stung by insects (labeled simply “anxiety”), cautious behaviors leading to decreasing a risk of sting incidents (“caution”), limitations in activities caused by attempts to avoid stings (“limitations”), discomfort caused by those limitations and the necessity to undertake cautious behaviors, as well as by associated feelings (“discomfort”), support received from parents with respect to coping with illness (“support”), and feeling of safety (“safety”). All the items and their associated response formats are presented in Table 2.

Stage 2) All these items were pretested in terms of how well they were understood by children and their relevance to the problems of the HVA children, based on a sample of 10 children - aged from 10 to 13 years - who were inhabitants of both towns and villages and were treated in the Department of Pediatrics. Pre-test wording of some items was corrected in accordance with the suggestions of the investigated children regarding how best to improve the understandability of the administered questionnaire and to make the meaning of such items more precise.

Stage 3) In May 2010, the 60-item questionnaire, containing a written consent to be signed by a parent or legal guardian, was mailed to 142 children born between the years of 1992 and 2000, who were undergoing diagnostic management in Polish-American Children Hospital, Kraków, Poland, for suspected HVA; 71 children returned the completed questionnaire by July 15.

Stage 4) For items in the domains labeled anxiety, caution and feeling of safety, a Likert response format based on a 5-point scale was established. For the other domains, such as limitations, discomfort or perceived support, which addressed behaviors or feelings related to the stimulus that might not have been reported by all the children, a quasi-Likert response format was created with the first option related to lack of this stimulus. In case of items in the domain of limitations, questions concerning “regret due to not doing something” had the following response format: 1. I can do this, 2. I don’t regret it, although I cannot do this, 3. I slightly regret I cannot do this, 4. I moderately regret I cannot do this, 5. I very much regret I cannot do this. Using Multiple Correspondence Analysis (MCA) for different sets of six items - each randomly selected from a separate domain – the relationship between quasi-Likert response options was examined [9]. The decision to treat options 1 and 2 (as showed in the example above) as the same level of limitations/discomfort/support was made if these categories were located close enough on the first dimension of the MCA solution, as presented on joint categories plot, in comparison to differences between other response options of the same item. Otherwise, these options were treated as different levels of measured latent variables.

Stage 5) For each domain, the matrix of correlations between its items was analyzed and items with the so-called salient correlations (i.e. correlations higher than 0.7) were excluded from the domain, so as to avoid including redundant items in the scale [10]. The exclusions were done according to the following criteria:

a) the variable with the highest number of salient correlations was excluded,

b) when two or more variables had the same number of salient correlations, the one with the highest salient correlation was excluded,

c) if the highest salient correlation described in point b) concerned two variables of interest, the variable with a higher second correlation with the items from the domain was excluded.

**Table 2:** Psychometric properties of all items, grouped into domains

		**A**	**B**	**C**	**D**	**E**
	**Anxiety**					0.63
A1	Anxiety about being stung	2.7	1.1	1	0.69	---
A2	Anxiety when seeing an insect that could sting you close to you	2.8	1.2	3	0.83	0.63
A3	Anxiety when seeing an insect that could sting you close to you when you are with your Parents	2.4	1.1	1	0.76	0.60
A4	Anxiety when seeing an insect that could sting you when you are with other adults (but not your parents)	2.5	1.1	3	---	---
A5	Anxiety about being stung when being at school	2.3	1.1	2	0.82	0.64
A6	Anxiety when seeing an insect that could sting you close to you when you are on your own;	3.0	1.2	7	---	---
A7	Anxiety when seeing an insect that could sting you close to you when you are with your peers (e.g. on your way back from school or doing a sport);	2.7	1.1	6	---	---
A8	Anxiety on being told that an insect that could sting you is flying behind you;	2.8	1.1	1	0.84	0.66
A9	Anxiety when an insect is so close to you that you have to ward it off	2.8	1.1	0	0.74	0.60
AA	Anxiety when seeing an insect that could sting you close to you when you are playing with your peers (at a friend’s birthday, a campfire or a school disco)	2.7	1.1	1	0.82	0.66
AB	Anxiety when seeing an insect that could sting you far away from you	2.4	1.1	0	0.75	0.60
	Possible answers: 1. not at all, 2. a bit, 3. moderately, 4. very much, 5. terribly					
	**Caution**					0.61
C1	Looking out for stinging insects	3.1	1.3	0	0.80	0.59
C2	Going away when seeing a stinging insect	3.7	1.2	0	0.84	0.66
C3	Avoiding places where can see a stinging insect	3.2	1.3	0	0.70	0.52
C4	Running away from a place where have seen a stinging insect	3.2	1.3	1	0.85	0.65
C5	Feeling like running away when seeing a stinging insect	3.2	1.4	2	---	---
C6	Warding off an insect flying so close that it could sting	2.6	1.2	0	0.33	---
C7	Wanting to hide when seeing a stinging insect close to someone	3.0	1.4	1	0.80	0.60
C8	Thinking about how to avoid being stung	3.0	1.2	0	0.81	0.63
	Possible answers: 1. never, 2. seldom, 3. sometimes, 4. often, 5. always					
	**Limitations**					0.68
L1	Regret due to not being able to do some things for fear of being stung	2.1	1.0	1	0.69	---
L2	Regret due to not being able to do some things out of doors for fear of being stung	2.3	1.1	5	---	---
L3	Regret due to not being able to do some things for fear of being stung when you are with your parents	2.2	1.1	4	---	---
L4	Regret due to not being able to do some things for fear of being stung when you are with other adults	2.1	1.1	5	---	---
L5	Regret due to avoiding places where insects appear	2.3	1.0	1	0.81	0.68
L6	Regret due to not being able to do some things for fear of being stung when you are at school	2.1	1.1	4	---	---
L7	Regret due to not being able to do some things for fear of being stung during school holidays, season breaks or other forms of time off school	2.4	1.2	5	0.86	0.67
L8	Regret due to not being able to do some things for fear of being stung when you are with your peers	2.3	1.2	1	0.86	0.68
L9	Regret due to not being able to do some things for fear of being stung when you are playing with your peers	2.4	1.1	4	---	---
LA	Regret due to not playing with your peers because you thought you could be stung at that time	2.1	1.3	1	0.80	0.69
	Possible answers: 1. I can do this, 2. I don’t regret it, although I cannot do this, 3. I slightly regret I cannot do this, 4. I moderately regret I cannot do this, 5. I very much regret I cannot do this.					
	**Discomfort**					0.68
D1	Discomfort due to looking out for stinging insects that could sting you;	2.6	1.2	3	---	---
D2	Discomfort due to looking out for insects when you are out of doors	2.7	1.2	2	0.81	0.66
D3	Discomfort due to looking out for insects that could sting you when you are with your parents	2.6	1.2	4	0.84	0.70
D4	Discomfort due to looking out for insects that could sting you when you are with other adults (not your parents)	2.7	1.2	6	---	---
D5	Discomfort due to avoiding places where insects appear	2.5	1.1	1	0.83	0.68
D6	Discomfort due to looking out for insects that could sting you when you are at school	2.2	1.2	1	0.84	0.67
D7	Discomfort due to looking out for insects that could sting you when you are on holiday	2.7	1.2	7	---	---
D8	Discomfort due to looking out for insects that could sting you when you are with your peers	2.5	1.2	3	0.83	0.70
D9	Discomfort due to warding off insects	2.5	1.1	0	0.64	---
DA	Discomfort due to looking for insects while playing with your peers	2.7	1.2	5	---	---
DB	Discomfort due to thinking about how to avoid being stung	2.7	1.1	2	0.81	0.66
	Possible answers: 1. I don’t do it, 2. I do it but I don’t feel any discomfort, 3. I feel slight discomfort, 4. I feel moderate discomfort, 5. I feel a lot of discomfort.					
	**Support**					0.58
S1	Talking to parents about being afraid of being stung	3.0	1.2	3	---	---
S2	Thinking that parents know very well about how afraid of being stung you are after talking to them about your fear	3.1	1.4	3	---	---
S3	Parents try to ease your fear of being stung when they are talking to you	3.2	1.5	3	0.86	0.63
S4	Talking to parents about risks that you face when you are stung	2.6	1.1	0	0.80	0.55
S5	Parents trying to calm you when they see an insect flying next to you	3.0	1.6	1	---	---
S6	Talking to parents about the way your peers treat you because of your fear of being stung	2.8	1.3	5	---	---
S7	After talking to your parents, feeling that they know well about the way your peers treat you because of your fear of being stung	2.8	1.6	1	0.82	0.59
S8	Talking to parents about the way your peers treat you because you try to avoid being stung	2.8	1.2	1	---	---
S9	After talking to parents, feeling that they know well about the way your peers treat you because you try to avoid being stung	2.7	1.6	1	0.75	0.53
	Possible answers: 1. never talk about that, 2. never, in spite talking about that, 3. seldom, 4. sometimes, 5. often,					
	**Feeling of safety**					0.45
F1	Thinking that you will be all right after having been being stung by an insect	3.0	1.0	0	−0.54	---
F2	Thinking that somebody will help after you have been stung	1.7	0.7	0	0.16	---
F3	Thinking that being stung puts your life at risk	2.2	1.0	0	0.75	0.47
F4	Thinking that bees/wasps are good insects	2.6	1.3	0	0.24	---
F5	Thinking that after being stung you will soon feel as well as before that	2.8	1.0	0	−0.63	0.41
F6	Thinking that after have been being stung you will be able to summon help, even if you feel very bad	2.1	0.8	0	−0.23	---
F7	Thinking that being stung puts somebody’s health at risk	2.3	1.1	0	0.75	0.48
F8	Thinking that bees/wasps only sting to defend themselves	2.1	1.0	0	0.00	---
F9	Thinking that somebody will help you if you feel very bad after you have been stung?	1.9	0.9	0	−0.02	---
FA	Thinking that something bad may happen after you have been stung	2.5	1.0	0	0.74	0.48
FB	Thinking that you will get soon feel well after you have been stung	2.6	1.0	0	−0.64	0.40
	Possible answers: 1. definitely yes, 2. rather yes, 3. neither yes nor not, 4. rather not, 5. definitely not					

The table presents: arithmetic means (column A) and standard deviations (column B) of particular item, number of salient correlations for each item (Column C), factor loadings of particular item obtained in PCA done for whole domain including this item (column D), and scalability coefficients H_i_ for particular items, and, in row presenting name of the domain, homogeneity coefficient H for the subscale measuring this particular latent variable, which included all items available in the domain at that stage of analysis (column E).

After each exclusion process, the number of salient correlations for the retained items was recalculated. The procedure was discontinued when there were no variables with salient correlations in the domain.

Stage 6) For each domain separately, the principal component analysis (PCA) was conducted and the variables which correlated with the first principal component lower than 0.7 were excluded, to retain items highly correlated with latent trait they were intended to measure [11].

Stage 7) Homogeneity of the subscales and scalability of the items were assessed separately for each domain - both these properties are measured with Loevinger scalability coefficient H [12]. These terms refer to the assumption that the items included in the scale create a hierarchical structure, i.e. if any person answers a particular item in the expected way, he responds to all the “easier” items (i.e. with a lower mean) in the same way. This ideal situation is described in the relevant literature as a perfect Guttman scalogram [13]. Homogeneity of the scale coefficient – further referred to as H – describes the extent of deviation from the perfect scalogram structure of the entire data structure (all the analyzed items), whereas scalability coefficients for i-th item – referred to as H_i_ – denotes deviation for particular items [13,14]. At this step of the analysis, the items with H_i_ lower than 0.5 were excluded.

Stage 8) 

a) if the subscale constructed with the remained items was characterized by H > =0.5, the pre-final version of the subscale measuring latent variable related to each domain included three items: the ones with the highest and the lowest mean, and the item with its mean closest to the mean of the previously selected items.

b) if the subscale constructed with the remained items was characterized by H < 0.5, the three items with the highest correlations with the first principal component (based on the results of stage 6) were selected to constitute the subscale.

Stage 9) Reliability of particular subscales in terms of their internal consistency was assessed: if the subscale included an item that caused an increase of reliability coefficient, after being removed from the scale, it was replaced by another item available at stage 8;

Stage 10) Multi-trait scaling was used and convergent and divergent validity of each item was assessed [15]. Convergent validity of the item was defined as its correlation with the score of the subscale to which the item belonged, after correction for overlap (i.e. after excluding that item from the total score of the subscale). Discriminant validity of the item was defined as its highest correlation of those with the scores of the subscales that did not include that item. An item was defined as divergently valid if its discriminant validity was lower than its convergent validity minus its standard error - that means that the item may be used as observed indicator of the latent trait it was intended to measure. If the item was not divergently valid, it was replaced by another item from its domain, which was available after stage 7, and the analysis was repeated beginning from stage 9. After necessary repetition of stages 9 and 10 for different sets of items, the items with the most optimal configuration of convergent and divergent validity as well as reliability of particular subscales were retained in the scale.

### Statistical analysis

Multiple Correspondence Analysis (MCA) was performed to check whether items for which responses were worded in quasi-Likert format scale behaved as ordinal variables, as did items with typical Likert response format [9].

All the correlations between the items included in a particular domain (computed at stages 5 and 9) were assessed using Kendall’s tau-b correlation coefficient.

The unidimensionality of the scales constructed to measure the particular domains were tested with principal component analysis (PCA).

The reliability of the subscales in terms of Classic Test Theory (CTT) with respect to their internal consistency was tested with the use of Cronbach alfa coefficient and with respect to Item Response Theory (IRT) with rho coefficient [8,13].

The homogeneity of the subscales and scalability of the items were tested using Mokken analysis procedure and measured with Loevinger H coefficient [12].

The null hypotheses were rejected at 0.05 level.

All the analyses were performed using SPSS 15 statistical package, except Mokken analysis, which was done with MSP 5 statistical software.

## Results

### Establishing response format for quasi-Likert items

**Figure 1:**
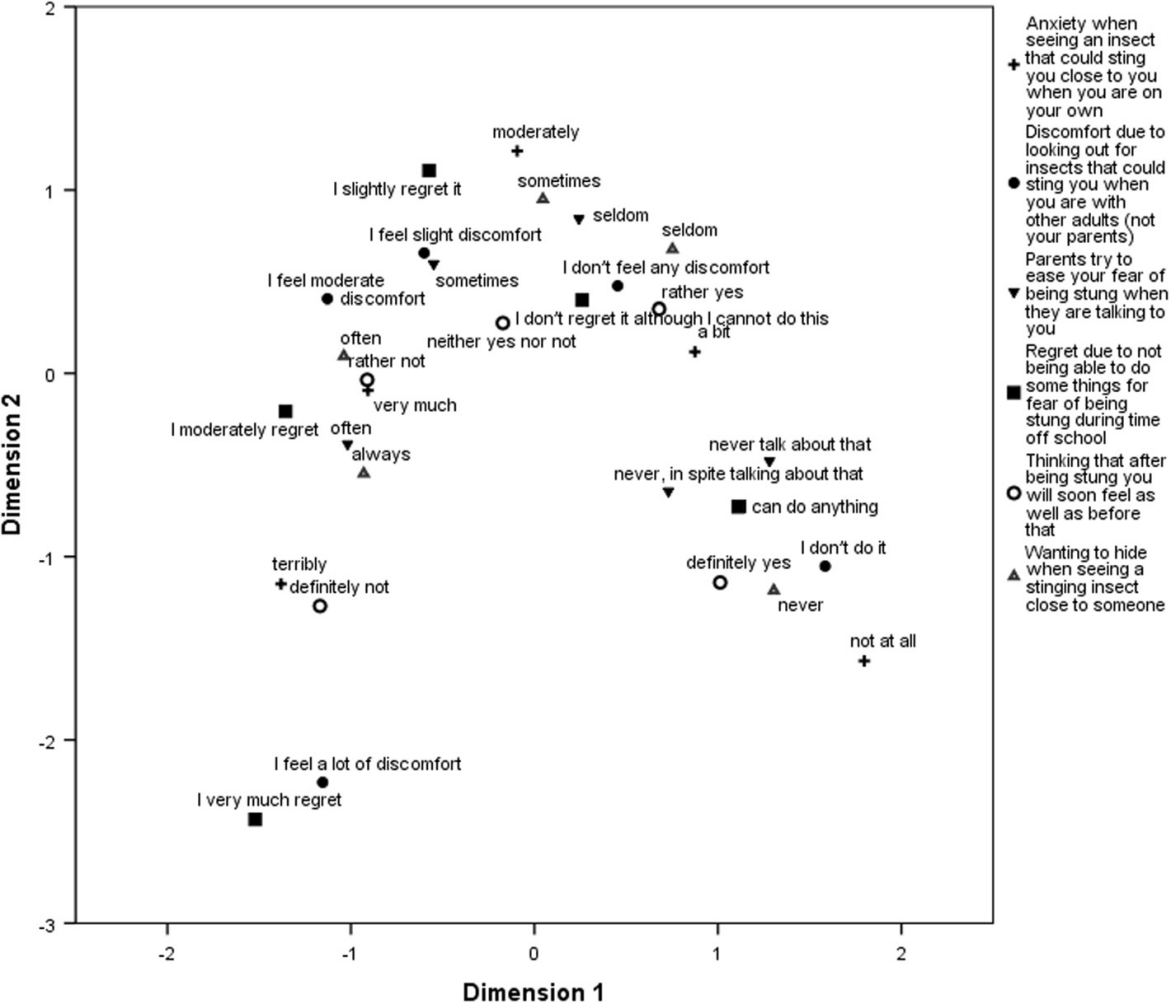
Joint category plot for set of one randomly selected item from each domain.

Figure 1 presents the results of MCA for one of several single items selected from each domain. The points in the figure represent centers of gravity of particular categories of the analyzed variables, i.e. the location of an average observation belonging to a given category. As all the variables are treated in this analysis as nominal ones, the ordinal hierarchy between particular categories is an effect of dependencies between categories of all variables, not their ordinal level of measurement. The arch shape observed in the figure is a consequence of the unimodal distribution of the majority of the analyzed variables [9]. Analysis of the 1^st^ dimension of the MCA solution (which explains a higher percentage of variance of the set of variables included in the analysis) shows that the first two options of items with quasi-Likert response format are located at a greater distance than other neighboring categories of the same items, for items included in the limitations (‘regret’ items) or discomfort domains. When analyzing items from the support domains, one may note that the distance between the first two options is similar to that between other neighboring options for that item. This may be interpreted that variables with quasi-Likert response format behave as typical Likert-scaled variables, and the option related, for instance, to lack of behavior causing limitations, and exhibiting behavior that causes no limitations, should be treated as different levels of limitations.

### Items selection

Table 2 presents the arithmetic means (column A) and standard deviations (column B) of all the items from the initial pool of 60 items grouped in the particular domains. Column C presents the number of salient correlations for each item. The analysis of correlation matrix performed for each domain separately provided a basis for excluding items with salient correlations, what led to decreasing the number of items available for creating particular subscales: 8 items for anxiety, 7 items for caution, 5 items for limitations, 7 items for discomfort, 4 items for support and 11 items for feeling of safety.

Principal component analysis conducted for each domain separately (factor loadings presented in Table 2, column D), allowed for deleting items whose correlation with the first principal components was lower than 0.7, what led to retaining 7 items for measuring anxiety, 6 items for caution, 4 items for limitations, 6 items for discomfort, 4 items for support and 5 items for safety.

Mokken analysis conducted separately for each domain showed that homogeneity coefficients H for the subscale including all the items from the domain exceeded 0.5 for all the domains except the domain indicating feeling of safety. Similarly, the H_i_ scalability coefficients for items available at that stage in the particular domains were higher than 0.5 for all the items, except all the items from the safety domain (Table 2, column E).

After applying Mokken analysis, the following variables were preselected to be included in the pre-final versions of particular subscales: A5, A8 and AA for measuring anxiety, C2, C4 and C8 for measuring caution, L5, L7 and LA for measuring limitations, D2, D8 and DB for measuring discomfort, S3, S4, and S7 for measuring support and F3, F7, FA and FB for measuring feeling of safety. After reliability analysis of the feeling of safety subscale, it became evident that the presence of the F3 item in the subscale caused removal of the FB item to trigger an increase of alpha coefficient value, so the F3 item was replaced by the F5 item. After an analysis of multi-trait matrix, the C8 variable occurred to be not divergently valid, as it correlated with the discomfort subscale, and thus it was replaced by C7, which, on the other hand, highly correlated with the anxiety subscale, and was in turn replaced by C1, which constituted the final version of the caution subscale. At that moment, none of the items caused a decrease of Cronbach alpha coefficient when removed from particular subscale; however, in case of the AA and A8 items, which had as high a correlation with caution and discomfort subscales, respectively, as with their own subscale, other variables available in this domain were considered to be included in the subscale, but their use always led to a marked decrease of Cronbach alpha coefficient, so the AA and A8 items were retained in the final version. The D8 item was retained in the subscale as it had a higher convergent validity when included in the subscale than other items left in the domain, and the S3 item was retained because it showed a higher correlation with the first principal component at stage 6 than the S9 item.

Table 3 presents psychometric properties of the developed subscales and their items. Column F of this table shows the values of Cronbach alpha coefficients of the final version of particular subscale (in the row containing the name of the subscale), and the value of alpha coefficient after removing the item from the subscale (in the row containing a particular item). Column G presents estimation of convergent validity of a particular item, computed as a correlation between the item and the summary score of the subscale to which it belongs, after correction for overlap. Column H contains the range of correlations between the item and the subscales which do not contain that item, whereas column I presents homogeneity coefficients, obtained from Mokken scaling analysis, for the subscales (in the row containing the name of a particular subscale) and scalability coefficients for their items (in the row containing a particular item) (Table 3).

**Table 3:** Psychometric properties of the developed subscales and their items^

		**F**	**G**	**H**	**I**
	**Anxiety**	**0.83**			**0.68**
A5	Anxiety about being stung when being at school	0.72	0.72	−0.36-0.59	0.71
AA	Anxiety when seeing an insect that could sting you close to you when you are playing with your peers	0.76	0.69	−0.41-0.64	0.69
A8	Anxiety on being told that an insect that could sting you is flying behind you;	0.79	0.66	−0.27-0.67	0.65
	**Caution**	**0.83**			**0.66**
C1	Looking out for stinging insects	0.78	0.66	−0.40-0.64	0.64
C2	Going away when seing a stinging insect	0.73	0.72	−0.27-0.56	0.69
C4	Running away from a place where have seen a stinging insect	0.77	0.67	−0.38-0.63	0.65
	**Limitations**	**0.82**			**0.68**
L7	Regret due to not being able to do some things for fear of being stung during school holidays, season breaks or other forms of time off school	0.73	0.70	−0.26-0.61	0.68
L5	Regret due to avoiding places where insects appear	0.74	0.72	−0.33-0.53	0.71
LA	Regret due to not playing with your peers because you thought you could be stung at that time	0.80	0.64	−0.30-0.60	0.66
	**Discomfort**	**0.83**			**0.84**
DB	Discomfort due to thinking about how to avoid being stung	0.79	0.66	−0.38-0.63	0.63
D2	Discomfort due to looking out for insects when you are out of doors	0.82	0.63	−0.37-0.54	0.61
D8	Discomfort due to looking out for insects that could sting you when you are with your peers	0.67	0.76	−0.34-0.65	0.71
	**Support**	**0.79**			**0.62**
S4	Talking to parents about risks that you face when you are stung	0.78	0.59	−0.42-0.58	0.59
S3	Parents try to ease your fear of being stung when they are talking to you	0.54	0.78	−0.26-0.67	0.70
S7	After talking to your parents, feeling that they know well about the way your peers treat you because of your fear of being stung	0.78	0.59	−0.14-0.49	0.57
	**Feeling of safety**	**0.72**	**-**		**-**
FA	Thinking that something bad may happen after you have been stung *	0.64	0.54	−0.27--0.04	-
F5	Thinking that after being stung you will soon feel as well as before that	0.67	0.49	−0.43--0.37	-
F7	Thinking that being stung puts somebody’s health at risk *	0.64	0.54	−0.30--0.12	-
FB	Thinking that you will get soon feel well after you have been stung	0.68	0.47	−0.34--0.21	-

The table presents: values of Cronbach alpha coefficients for particular subscales in row containing name of the subscale, and value of alpha coefficient after removing the item from the subscale, in row containing particular item (Column F). Column G presents estimation of convergent validity of particular item. Column H contains the range of correlations between the item and subscales which do not contain that item. Column I presents homogeneity of the scale and scalability coefficients for particular items.

* Item responses need to be reversed before scaling.

^ Warning: Presented wording of items is only a provisional translation of Polish version of the scale, prepared for presentation in this paper, not a validated English version.

### Subscales divergent validity

Correlations between particular subscales (except feeling of safety) ranged from 0.3 to 0.6, suggesting a weak to moderate relationship between particular dimensions of children’s HRQoL and supporting the thesis that the extracted dimensions of HRQoL do not overlap. Correlations of the feeling of safety subscale with other dimensions ranged from −0.16 (not significant with respect to support and limitations) to −0.31 (Table 4).

**Table 4:** Correlations between subscales

	**Anxiety**	**Caution**	**Limitations**	**Discomfort**	**Support**	**Safety**
Anxiety	1					
Caution	0.43	1				
Limitations	0.47	0.36	1			
Discomfort	0.50	0.53	0.58	1		
Support	0.46	0.29	0.49	0.48	1	
Safety	−0.31	−0.29	−0.19 (ns)	−0.26	−0.16 (ns)	1

### Reliability and scalability of the subscales

Reliability of all the subscales measured with Cronbach’s alpha coefficient was higher than 0.8, except the support and feeling of safety subscales, for which alpha coefficient equaled 0.79 and 0.72, respectively (Table 5). None of the scale items caused an increase of reliability coefficient value when being removed from its subscale (Table 3). Reliability of the subscales measured with rho coefficient was also higher than 0.8 for all the subscales, except the safety subscale, which was finally not evaluated in terms of IRT. Homogeneity coefficient H for particular subscales reached values higher than 0.6, whereas particular items were characterized by Hi coefficients higher than 0.5 (Table 5).

**Table 5:** Reliability and scalability coefficients for the subscales

**Subscale\Coefficient**	**alpha**	**rho**	**H**	**H**_**i**_
Anxiety	0.83	0.84	0.68	0.65 - 0.71
Caution	0.83	0.83	0.66	0.64 - 0.69
Limitations	0.82	0.84	0.68	0.66 - 0.71
Discomfort	0.83	0.85	0.84	0.61 - 0.71
Support	0.79	0.82	0.62	0.57 - 0.70
Safety	0.72	---	---	---

## Discussion

This paper presents the development of a HRQoL scale for children and adolescents with HVA. Our efforts were oriented towards developing the short scale (up to 5 items each) measuring 6 dimensions of HRQoL of children with HVA, taking into account reliability of the chosen sets of items and correlation patterns of selected items and other subscales using multi-trait matrix analysis [15]. The scale items were formulated with no evident referral to illness the scale is dedicated to, as assuming that such feelings as anxiety about insects, discomfort caused by such anxiety, feeling of safety with respect to possible stings and their consequences, or finally, behaviors leading to avoiding stings are related not only to the diagnosis of HVA, but also exist in a healthy population and their extent is associated with psychological characteristics of an individual.

The tool consisting of six subscales, selecting the items separately for each subscale; finally proved in multi-trait method that most of the items correlated stronger with other items belonging the same subscale than with other subscales. One item had lower correlation with its own subscale than with the other subscales, and a few items having a correlation with other subscales within one standard error of the correlation with their own subscale. In spite of those objections concerning divergent validity of the items, correlation between any two subscales did not reach 0.6, what is distinctly lower than the 0.7 threshold usually considered as indicating redundancy between variables [10].

Correlations between the created subscales were weaker than those obtained for the Polish adaptations of VQLQ for adolescents, and stronger than were those seen in the Polish version of VQLQ for children, (except the correlation between anxiety and caution, which was weaker than in the Polish VQLQ for children). That seems to prove that the new scale better distinguishes between the measured theoretical constructs as compared to the previously used scales [3,4].

In both Polish adaptations of VQLQ, limitations were measured with one question with broad interpretation by respondent. Hence in created scale, the assessment of limitations was based on three particular criteria, not on direct question “how limited do you feel”, as authors were afraid that such a question might not be well understood by younger children.

Although created scales for measuring particular dimensions of HVA patients’ HRQoL were short, the internal consistency of all the subscales, except feeling of safety, was close to 0.8, whereas Cronbach alpha for this particular subscale exceeded 0.7, what is considered as minimum for group comparisons [16]. Also at least as high reliability as in case of the anxiety and caution subscales of the Polish adaptations of VQLQ for children and adolescents was obtained. These adaptations measured anxiety with reliability of at least 0.79, whereas in this paper the value of 0.83 was obtained. In case of the caution subscale, also alpha equal to 0.83 was obtained, whereas in the adaptation of VQLQ for adolescents alpha reached 0.80 and only 0.53 in the adaptation for children [3,4]. In case of discomfort measurement, a lower value of internal consistency coefficient (0.83) was obtained, than in both children and adolescent adaptations of VQLQ; however, in these versions of the index, the discomfort subscale consisted of 7 items, with some of them correlating higher than 0.7. If these items were removed, it would lead to only three items remaining in the subscales, with alpha coefficient for the subscale equal to 0.87 [3,4,17].

In psychometric properties of the subscales estimated in terms of IRT, reliability coefficients were even higher than those measured in terms of CTT. Rho reliability coefficients were higher than 0.8 for all five subscales evaluated in terms of that theory. In the analysis of the feeling of safety subscales as a hierarchical tool, its reliability would equal 0.65. Scalability coefficients for five developed subscales were also very high - over 0.6 for all these subscales, and for all the items of four of these subscales - and they markedly exceeded the threshold of 0.5 usually considered to indicate scales with a strong hierarchical relationship [13]. These values cannot be compared with other scales measuring HRQoL in HVA patients, as they were not evaluated in terms of IRT [1,3,4].

However, we were confronted with certain difficulties which forced us to make decisions that may have affected the value of the developed instrument. The most influential of this decisions was our choice to not use impact methodology-which is often used in case of rare diseases (such as HVA, asthma or food allergy) [1,18-20] - and instead use a methodology rooted in psychological research on traits of personality, which is often used in HRQoL research [21-23]. The most important consequence of omitting the use of factor analysis during the development of new scale using impact method is the resulting lack of a thorough and proper assessment of multidimensionality of the created tool, what makes its score interpretation unclear [3,4]. Another consequence of using impact methodology is that selection of the items is based on different properties (importance and relevance) than will be interpreted in the studies using this scale (scores of the items).

One of the most popular response formats in HRQoL and health status measurement is the 5-point Likert scale, which does not include the option “not applicable [24]”. This is necessary in the situation when a researcher wants to ask about discomfort related to avoiding places of high insects exposure, while in patients not avoiding such places, the question is not applicable. Hence it leads to a lower response rate in comparison to other items, while computing the score of the subscale based on equal number of responses from each respondents gives more reliable results. Including such option arises the problem whether the situation when someone does not avoid insect-abounding places (and therefor feels no discomfort) evaluate as equal to the answer that the someone feels no discomfort due to avoiding such places. We could assume so, but on the other hand, avoiding such places requires some effort which is not consciously perceived as causing discomfort. MCA showed that, in the space defined by variables coming from different domains, the above-mentioned answers are located at different coordinates [25]. It seems that the difference between these two options is similar to that observed between other answer options, and should be treated as different levels of the measured latent variable. A similar decision was reached with respect to treatment of limitations and support items.

The main limitation of this study is that the development of a measurement tool with the use of psychometric methodology demands a proper sample size, especially for application of PCA, which requires at least 5 observations per each variable included in the analyzed set of items. To create a scale of about 20 items, it is required to get the answers from least 100 children [11]. Though we collected a sample of 142 children aged 10 to 18 years, who were diagnosed to HVA in Polish-American Children’s Hospital in Krakow, they were mostly eligible by mail only, due to their place of residence (up to 250 km from Krakow). The questionnaires were sent to all the available children with the history of insect sting reaction (both systemic and large local reactors), aiming to develop a scale useful for the entire sample of young HVA patients, irrespectively of their Mueller grade of allergic reaction. Response rate in our study achieved 50%, what is in line with the other mail surveys, also large epidemiological studies. Sociologists consider this level as providing reliable and valid data [26,27]. Additionally, using a mail survey did not guarantee the same conditions at the moment of filling the questionnaire for all respondents; on the other hand, these conditions were better reflecting their everyday environment, what could render the results of measurement reliable.

Because the sample size was not sufficient to provide analysis of multidimensionality of the scale with the use of PCA, we tried to reach the goal by some indirect means, namely by conducting multi-trait scaling. Additionally, we decided to relax the condition of distinguishing between convergent and divergent validity from two standard errors, as proposed by Fayers, to one standard error, since standard error size depends on sample size, and in our small sample, this would lead to treating many items as not divergently valid [15].

Due to the reason that we had to develop the scale using the entire available sample, the cross-sectional validation was not provided, though it would allow for confirming the psychometric properties of the scale in patients not involved in the development process. As HVA is a rare disease, a number of patients qualified every year to treatment is rather small (about 10–15 children per year even in large medical centers with a catchment area apx. 300 km in diameter), gathering a sample of at least 100 patients required to conduct exploratory factor analysis (or at least 200 patients needed to conduct confirmatory factor analysis) would be very time-consuming.

## Conclusions

The presented scale seems to be a valid and reliable tool for measuring HRQoL in HVA children and adolescents, and its use will provide deeper insight into this matter and may be useful in everyday clinical practice. Polish validated version of CHVAQoLS is enclosed in Additional file 1.

## Abbreviations

HRQoL: Health-related quality of life; VQLQ: Vespid quality of life questionaire; HVA: Hymenoptera venom allergy; PCA: Principal component analysis; MCA: Multiple corespondence analysis; CTT: Classical test theory; IRT: Items response theory.

## Competing interests

The authors declare no competing interests.

## Authors’ contributions

EC-J contributed to design of the study and its coordination, data collection and drafted the manuscript. PB contributed to design of the study and data collection, performed the statistical analysis and drafted the manuscript. BTA helped to draft the manuscript and revising it critically for important intellectual content. GL helped to draft the manuscript and revising it critically for important intellectual content. JJP helped to draft the manuscript and revising it critically for important intellectual content. All authors read and approved the final manuscript.

## Supplementary Material

Additional file 1 Polish validated version of CHVAQoLS. Click here for file
