# Supplementary material for: Development of children’s hymenoptera venom allergy quality of life scale (CHVAQoLS)
Source: Clin Transl Allergy. 2013 Aug 1;3:25. doi: 10.1186/2045-7022-3-25 (PMC3750314; doi:10.1186/2045-7022-3-25)
Supplement: Additional file 1 — Polish validated version of CHVAQoLS. [file 2045-7022-3-25-S1.doc]

**Skala jakości życia dzieci z alergią na jad owadów błonkoskrzydłych**

Alergia na jad owadów błonkoskrzydłych (pszczoły, osy lub szerszenie) może objawiać się u Ciebie w różnej postaci. Poniższe pytania mają ocenić, jak duży wpływ na Twoje życie ma alergia, na którą chorujesz. Odpowiedz na nie zaznaczając kółkiem odpowiedzi, które najlepiej opisują Twoje zachowywania i odczucia dotyczące sytuacji opisanych poniżej.

**UWAGA:** W każdym pytaniu podkreślone słowo: owad, owada, itd., oznacza owada, na jad którego jesteś uczulony/a: tylko pszczołę, tylko osę (ew. szerszenia), albo „osę lub pszczołę”, jeśli jesteś uczulony/a na jad obu owadów.

Imię i nazwisko……………………………………………………. Płeć: M K

Data urodzenia………..……..……. Data wypełnienia ankiety…….……..………….....

Owad uczulający…..………………… Data ostatniego użądlenia…..…………...............

**1. Jak często rozglądasz się czy w pobliżu Ciebie są owady, które mogą Cię użądlić?**

a. nigdy

1. rzadko
2. czasami
3. często
4. zawsze

**2. Czy myślisz, że po użądleniu przez owada może stać Ci się coś złego?**

1. na pewno tak
2. raczej tak
3. nie wiem
4. raczej nie
5. na pewno nie

**3. Jak bardzo boisz się użądlenia, kiedy jesteś w szkole?**

a w ogóle się nie boję

b trochę się boję

c. średnio się boję

d. bardzo się boję

e. strasznie się boję

**Aby uchronić Cię przed użądleniem przez owada, Rodzice lub lekarz mogą zabronić Ci robić pewne rzeczy.**

**4. Jak silny żal czujesz, z tego powodu, że będąc na wakacjach, feriach, lub w inny sposób spędzając czas wolny od szkoły, nie możesz robić pewnych rzeczy aby uniknąć użądlenia,?**

1. wolno mi robić wszystko
2. nie czuję żalu, chociaż nie mogę robić pewnych rzeczy
3. czuję lekki żal
4. czuję średni żal
5. czuję silny żal

**5. Jak bardzo przeszkadza Ci to, że myślisz o tym jak uniknąć użądlenia?**

a. nie myślę o tym jak uniknąć użądlenia

b. myślę o tym jak uniknąć użądlenia ale wcale mi to nie przeszkadza

c. trochę mi to przeszkadza

d. średnio mi to przeszkadza

e. bardzo mi to przeszkadza

**6. Jak często rozmawiasz z Rodzicami o tym, czym może grozić Ci użądlenie przez owada?**

a. nigdy

b. rzadko

c. czasami

d. często

e. przy każdej okazji

**7. Czy myślisz, że po użądleniu przez owada szybko będziesz czuł się tak dobrze jak przed użądleniem?**

1. na pewno tak
2. raczej tak
3. nie wiem
4. raczej nie
5. na pewno nie

**8. Jak często widząc owada, który(a) może cię użądlić, odchodzisz z miejsca, w którym lata owad?**

- 1. nigdy
  2. rzadko
  3. czasami
  4. często
  5. zawsze

**9. Jak bardzo boisz się, gdy bawiąc się z rówieśnikami (np. na urodzinach kolegi/koleżanki, na ognisku, na dyskotece) widzisz obok siebie owada, który może Cię użądlić?**

a w ogóle się nie boję

b trochę się boję

c. średnio się boję

d. bardzo się boję

e. strasznie się boję

**10. Jak silny żal czujesz z tego powodu, że unikasz miejsc, w których pojawiają się owady?**

1. wolno mi chodzić wszędzie
2. nie czuję żalu, chociaż nie chodzę w pewne miejsca
3. czuję lekki żal
4. czuję średni żal
5. czuję silny żal

**11. Jak bardzo przeszkadza Ci to, że rozglądasz się za owadami, gdy jesteś na świeżym powietrzu?**

a. nie rozglądam się za owadami

b. rozglądam się za owadami, ale wcale mi to nie przeszkadza

c. trochę mi to przeszkadza

d. średnio mi to przeszkadza

e. bardzo mi to przeszkadza

**12. Jak często Rodzice rozmawiając z Tobą starają się zmniejszyć Twój lęk przed użądleniem?**

a. nigdy, bo nie rozmawiamy na ten temat

b. nigdy, chociaż rozmawiamy na ten temat

c. rzadko

d. czasami

e. często

**13. Czy myślisz, że użądlenie przez owada jest groźne dla Twojego zdrowia?**

1. na pewno tak
2. raczej tak
3. nie wiem
4. raczej nie
5. na pewno nie

**14. Jak często widząc obok siebie owada, który może Cię użądlić, uciekasz z miejsca w którym lata ten owad?**

- 1. nigdy
  2. rzadko
  3. czasami
  4. często
  5. zawsze

**15. Jak bardzo boisz się gdy ktoś Ci powie, że za Tobą lata owad, który może Cię użądlić?**

a w ogóle się nie boję

b trochę się boję

c. średnio się boję

d. bardzo się boję

e. strasznie się boję

**16. Jak silny żal czujesz, gdy nie bawisz się z rówieśnikami, dlatego że myślałeś/aś, że w czasie zabawy możesz zostać użądlony/a?**

1. wolno mi robić wszystko
2. nie czuję żalu, chociaż nie mogę robić pewnych rzeczy
3. czuję lekki żal
4. czuję średni żal
5. czuję silny żal

**17. Jak bardzo przeszkadza Ci to, że będąc z rówieśnikami (np. wracając ze szkoły, uprawiając sport itp.) rozglądasz się za owadami, które mogą Cię użądlić?**

a. nie rozglądam się za owadami

b. rozglądam się za owadami, ale wcale mi to nie przeszkadza

c. trochę mi to przeszkadza

d. średnio mi to przeszkadza

e. bardzo mi to przeszkadza

**18. Jak często po rozmowie z Rodzicami o Twoim lęku przed użądleniem, wydaje Ci się, że Rodzice dobrze wiedzą jak z powodu Twojego lęku przed użądleniem traktują Cię rówieśnicy?**

a. nigdy, bo nie rozmawiamy na ten temat

b. nigdy, chociaż rozmawiamy na ten temat

c. rzadko

d. czasami

e. często

**19. Czy myślisz, że po użądleniu przez owada znowu szybko będziesz czuł/a się dobrze?**

1. na pewno tak
2. raczej tak
3. nie wiem
4. raczej nie
5. na pewno nie

**DZIĘKUJEMY ZA UDZIELENIE DOKŁADNYCH ODPOWIEDZI I POŚWIĘCONY CZAS**
